# Initial Outcomes and Survival of Out-of-Hospital Cardiac Arrest: EuReCa Serbia Multicenter Cohort Study

**DOI:** 10.7759/cureus.18555

**Published:** 2021-10-06

**Authors:** Srdjan S Nikolovski, Aleksandra D Lazic, Zoran Z Fiser, Ivana A Obradovic, Suzana S Randjelovic, Jelena Z Tijanic, Violetta I Raffay

**Affiliations:** 1 School of Medicine, University of Belgrade, Belgrade, SRB; 2 Emergency Department, Clinical Center of Vojvodina, Novi Sad, SRB; 3 Emergency Department, Municipality Institute for Emergency Medicine Novi Sad, Novi Sad, SRB; 4 Anesthesiology, Resuscitation and Intensive Care Department, Hospital Sveti Vracevi, Bijeljina, BIH; 5 Emergency Medical Service, University Clinical Center Kragujevac, Kragujevac, SRB; 6 Emergency Medicine, Emergency Medical Service, Kragujevac, SRB; 7 School of Medicine, European University Cyprus, Nicosia, CYP

**Keywords:** automatic external defibrillator, survival-to-discharge, return of spontaneous circulation, eureca, cardiopulmonary resuscitation, out-of-hospital cardiac arrest

## Abstract

Introduction

Although the global survival rate of patients after out-of-hospital cardiac arrest (OHCA) has increased in the previous years, there still remain significant multifactorial public health challenges with many important aspects influencing the overall survival rate of these patients. The objective of this article is to analyze basic epidemiological parameters of OHCA in Serbia and to evaluate the influence of pre-hospitalization factors on the survival of OHCA patients.

Methods

Data on OHCA within the EuReCa Serbia Registry was collected according to the EuReCa Study protocol during the period October 1, 2014 - December 31, 2019, and included basic demographic data of the patients, data related to OHCA prior to hospital arrival, as well as data regarding subsequent hospitalization.

Results

The study included 6,266 EuReCa events (54% males), with a median age of 73 years [interquartile range (IQR) 63-82]. Cardiac arrest was witnessed in 3,111 out of 6,266 cases (49.6%), of which 2,725 cases (87.6%) were witnessed by bystanders and 286 cases (12.4%) by the emergency medical service (EMS) team. Resuscitation measures were attempted in 2,097 of 3,111 (67.4%) witnessed OHCA cases. Bystander cardiopulmonary resuscitation (CPR) was initiated in 288 cases within the bystander-witnessed group of 2,725 cases (10.6%). An initial shockable rhythm was detected in 323 out of 3,111 witnessed cases (10.4%). Any return of spontaneous circulation (ROSC) prior to hospital arrival was observed in 441 out of 2,097 cases where CPR was initiated (21.0%). Within the group of 2,097 events where CPR was initiated, in 287 cases the patient was transported to the hospital with ROSC (13.7%). An automated external defibrillator (AED) was used by bystanders in three cases. The collapse in locations other than the place of residence [p < 0.01; odds ratio (OR) 3.928], attempt to initiate CPR by a bystander (p < 0.01; OR 2.169), and presence of initial shockable rhythm (p = 0.01; OR 2.070) were observed as significant predictors of any ROSC in OHCA patients. Out of 287 patients hospitalized with ROSC, 54 (18.8%) were discharged alive.

Conclusion

Collapse outside of residence place, bystander CPR initiation, and initially detected shockable rhythm are important predictors of ROSC prior to hospital arrival and overall survival. Key factors of CPR-providing performance observed in this study were witnessing OHCA, CPR initiated by a bystander, presence of initial shockable rhythm, and any ROSC prior to hospital arrival.

## Introduction

It is generally known that cardiac arrest (CA) in the out-of-hospital environment is a multifactorial problem [1-4] that can be analyzed from several aspects. Despite the great efforts made towards the improvement of the application of emergency healthcare interventions in patients with out-of-hospital cardiac arrest (OHCA), there are still shortcomings in different areas of managing these patients.

One of the encouraging parameters is that survival of patients with OHCA is slowly improving over time [4,5] and in Europe, high survival rates have been described [6,7]. These findings can be largely attributed to the efforts on the advancement of all segments of proper emergency medical service (EMS) functioning, and perhaps mostly on improving implementation of chain of survival.

In Serbia, management of CA patients is based on the active involvement of the general population which precedes EMS system engagement. Therefore, the objective of this article is to analyze basic epidemiological parameters of OHCA in Serbia, to describe the involvement of bystanders and the outcome of management of OHCA patients in terms of the occurrence of return of spontaneous circulation (ROSC), as well as to evaluate the influence of pre-hospitalization factors on survival of OHCA patients.

Part of the results in this article was previously presented at the Bosnian-Herzegovinian American Academy of Arts and Sciences (BHAAAS) conference 12th Days of BHAAAS on June 24, 2021.

## Materials and methods

The study included epidemiological data on OHCA collected through the questionnaire of the European Resuscitation Council's (ERC) EuReCa ONE study. The criterion for admission was OHCA observed by EMS mobile teams. Pediatric patients were also included in the analysis, as well as patients with CA of non-cardiac causes (including trauma). The collected database consisted of the information defined by the unique EuReCa ONE study protocol during the period October 1, 2014 - December 31, 2019. Upon completion of each questionnaire, the data was entered into a unique electronic database in each investigation center and subsequently into the centralized database.

EuReCa ONE is an international, prospective, multicenter study of survival of patients (epidemiology, treatment, and outcomes) who experienced OHCA in Europe. The study is registered with "clinicaltrials.gov" (Registration Number: NCT02236819) by the ERC.

In this study, data on the age of the patients, gender, etiology of OHCA, place of OHCA, presence of a witness, bystander cardiopulmonary resuscitation (CPR), the occurrence of any form of ROSC prior to hospital arrival, use of an automated external defibrillator (AED), presence of initial shockable rhythm, and data regarding subsequent hospitalization (survival to discharge and 30-day survival rate) were analyzed.

Data was collected from 40 municipalities in Serbia representing 48.7% of the Serbian population. The municipalities were enrolled in the study on a voluntary basis after sending enrollment invitations to the randomly selected sample of municipalities with developed local EMS systems.

Statistical analysis of data was performed using IBM Corp. Released 2019. IBM SPSS Statistics for Windows, Version 26.0. Armonk, NY: IBM Corp and GraphPad Prism version 8.0.0 for Windows, GraphPad Software, San Diego, California USA software packages. Normality was assessed by calculating distribution deviation (skewness and kurtosis z-values), as well as by applying Kolmogorov-Smirnov with Lilliefors significance correction and Shapiro-Wilk normality tests. That preparatory step was followed by descriptive statistical procedures. Analytic statistical steps included the Mann Whitney U test which determined the association between variables, as well as linear, multiple, and binary logistic regression analyses adjusted for investigated factors and demographic characteristics which analyzed relationships within data. Survival was assessed by using the log-rank test and Cox proportional hazard regression analysis.

## Results

The analysis was performed on 6,266 EuReCa events within the Serbian OHCA registry (232.1/100,000 per year). Males represented 54.2% of the study population. The majority of patients (81.6%) were older than 60 years of age with a median value of 73 years [interquartile range (IQR) 63-82]. Fifty-seven patients in the registry (0.9%) were under 18 years of age. In 5,075 cases (81.0%), the location of OHCA was the patient's place of residence.

Etiology data of EuReCa events was collected in 3,922 cases with the distribution as follows: cardiac etiology (2,900/3,922 cases; 74.0%), trauma (178/3,922 cases; 4.5%), submersion (8/3,922 cases; 0.2%), respiratory disease (131/3,922 cases; 3.3%), and other non-cardiac causes (705/3,922 cases; 18.0%). In 2,344 cases within the registry etiology was not defined.

**Figure 1:**
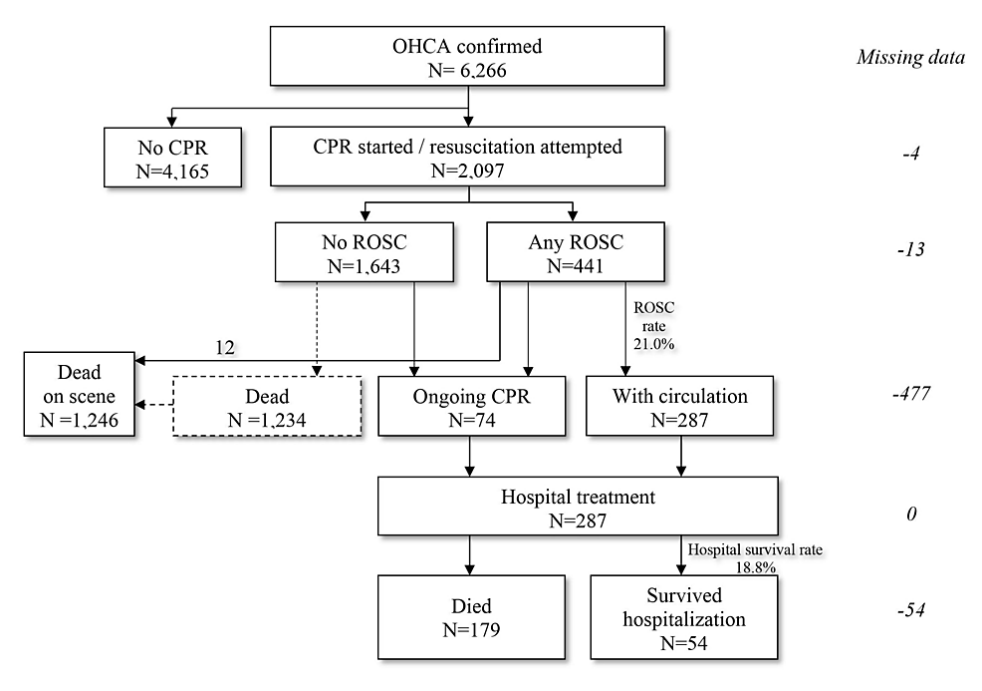
Flow diagram of all EuReCa events in the study CPR - cardiopulmonary resuscitation, OHCA - out-of-hospital cardiac arrest; ROSC - return of spontaneous circulation

Cardiac arrest was witnessed in 3,111/6,266 cases (49.7%), of which 2,725/3,111 cases (87.6%) were witnessed by bystanders and by the EMS mobile team in 286 cases (12.4%). Resuscitation was initiated in 2,097/3,111 cases (67.4%) which represents the incidence of 77.7/100,000 per year. Bystander CPR was initiated in 288 patients within the group of 2,725 bystander-witnessed cases (10.6%). An initial shockable rhythm was observed in 323/3,111 cases (10.4%). Any ROSC prior to hospital arrival was observed in 441/2,097 cases (21.0%). In 4,165 patients resuscitation measures were not attempted due to the following reasons: the presence of irreversible signs of death, the presence of do-not-attempt-resuscitation order, and the absence of medical indications for CPR initiation. Any form of ROSC in patients with OHCA of any cause was present in 441 cases out of 2,097 patients with initiated CPR (21.0%), i.e. in 7.0% in a group of all EuReCa events. AED was used by bystanders in only three cases. Considering shockability of initially detected heart rhythms, shockable rhythm was detected in 339 in 2,097 witnessed EuReCa events (16.2%). Out of 361 patients transported to the nearest hospital, 287 were hospitalized (Figure 1).

**Figure 2:**
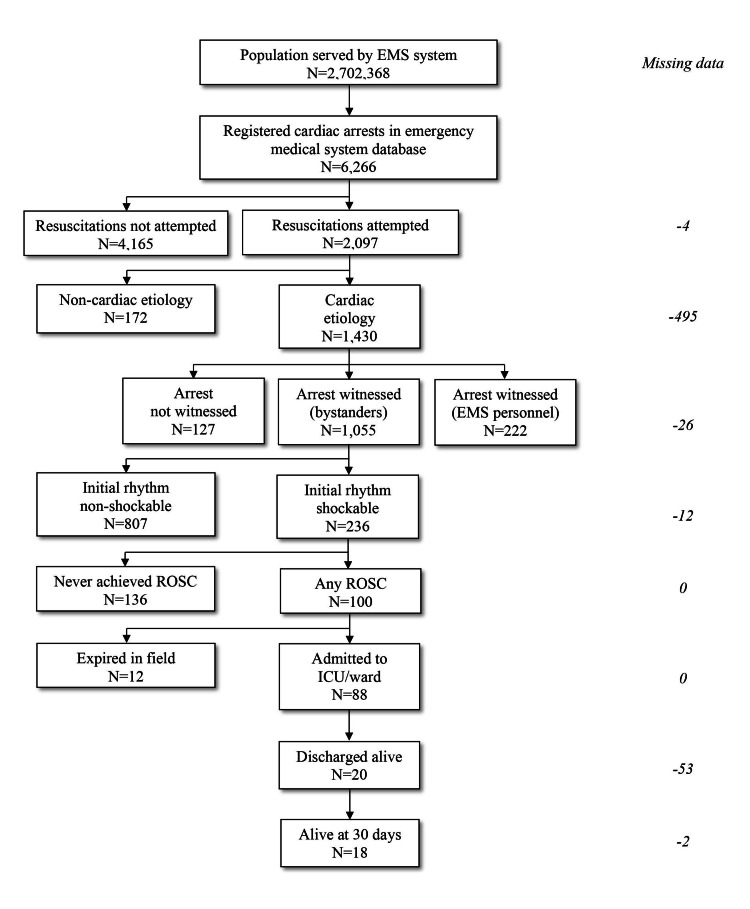
Flow diagram with number of Utstein-event cases EMS - emergency medical service; ROSC - return of spontaneous circulation; ICU - intensive care unit

Out of 2,097 events where CPR measures were initiated, cardiac etiology took part in 1,430 cases (68.2%). Out of those cases, a bystander was a witness in 1,055/1,430 cases (73.8%), where initial shockable rhythm was detected in 236/1,055 cases (22.4%). Within this group of Utstein events (cardiac etiology, bystander-witnessed collapse, initiated CPR, shockable initial rhythm observed), ROSC was achieved in 100/236 cases (42.4%). Twelve patients eventually died prior to hospitalization while 88 were hospitalized with ROSC. Twenty patients out of 100 with any ROSC (20.0%) were discharged alive (Figure 2) (Table 1).

**Table 1:** Patient and system factors in the group of CPR-attempted OHCA patients CPR - cardiopulmonary resuscitation; OHCA - out-of-hospital cardiac arrest; ROSC - return of spontaneous circulation

Variable	Value
Mean age (years)	70.8
Male gender (%)	65.4
Medical/cardiac cause (%)	79.3
Traumatic cause (%)	1.8
Location: residence (%)	74.1
Telephone CPR (%)	4.7
Collapse witnessed (%)	49.6
Bystander CPR (%)	10.6
Shockable rhythm (%)	16.2
ROSC (%)	21.0

**Figure 3:**
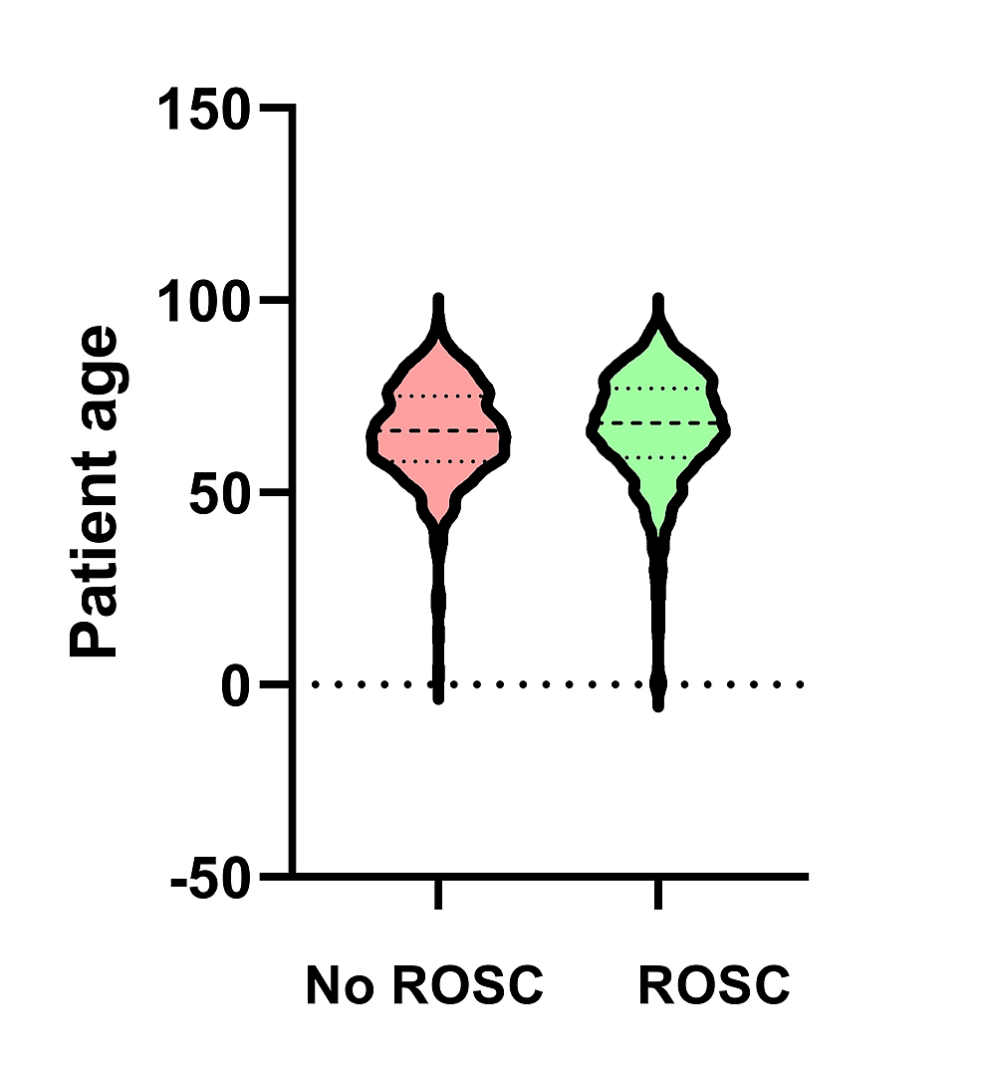
Age distribution in patients with and without return of spontaneous circulation after out-of-hospital cardiac arrest ROSC – return of spontaneous circulation

Within a group of 2,097 witnessed events, the Mann-Whitney-U test indicated that the patients who achieved any form of ROSC (Med=68; IQR 58-75) were significantly older compared to the patients who did not achieve any ROSC (Med=66; IQR 59-77), U=337,426.5, p=0.03 (Figure 3).

Regression analysis reported the occurrence of OHCA outside place of residence [p˂0.01; odds ratio (OR) 3.928], attempt to initiate CPR by a bystander (p˂0.01; OR 2.169), as well as the presence of initial shockable rhythm (p=0.01; OR 2.070) as significant predictors of ROSC prior to hospitalization in OHCA patients.

In the group of 287 hospitalized OHCA survivors of any cause receiving CPR at the collapse location median value of age was 66 years (IQR 59-75). Of all hospitalized OHCA patients, 62 (21.6%) received bystander CPR at the collapse location prior to hospitalization, out of which, chest compressions only were attempted in 33 cases, and compression-ventilation combination in 29 cases. Dispatcher assistance was used in 39 cases (62.9% of all hospitalized OHCA patients receiving any form of bystander CPR). In the group of hospitalized patients who received any form of CPR, initial shockable rhythm was detected in 134 cases.

**Figure 4:**
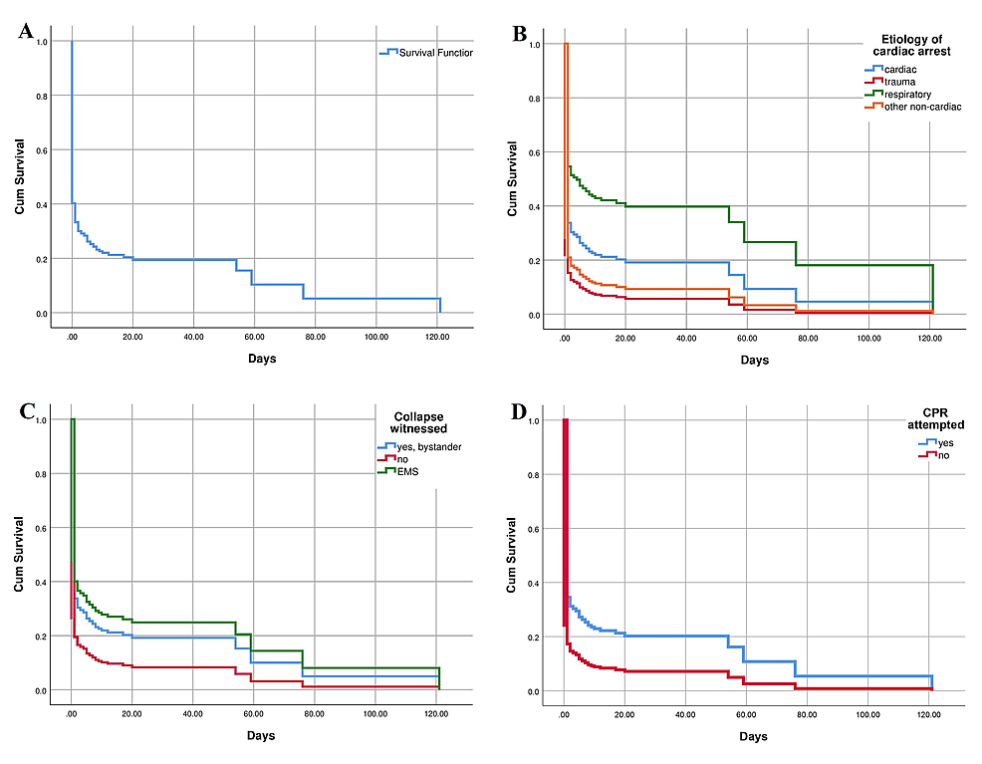
Kaplan-Meier curve displaying overall survival (A), survival related to etiology of cardiac arrest (B), survival related to witnessing of cardiac arrest (C), and survival related to attempt of cardiopulmonary resuscitation (D) in hospitalized patients after out-of-hospital cardiac arrest EMS - emergency medical service; CPR - cardiopulmonary resuscitation;

**Figure 5:**
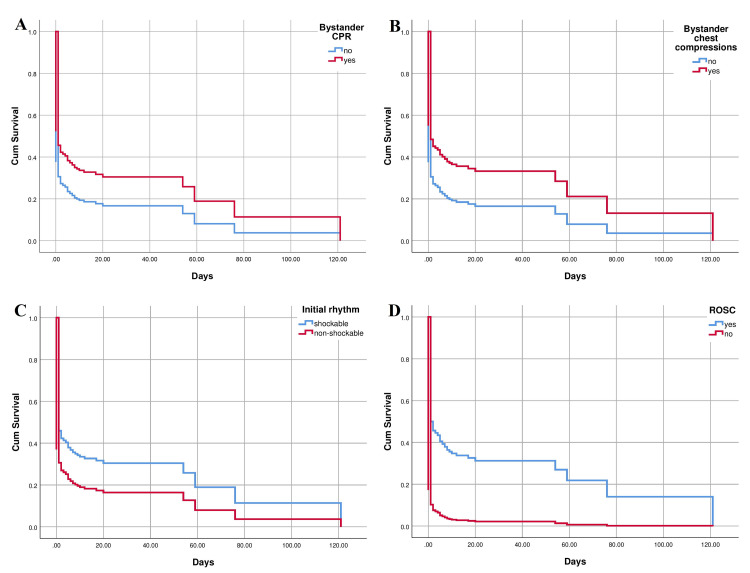
Kaplan-Meier curve displaying survival related to presence of bystander cardiopulmonary resuscitation (A), survival related to chest compressions only applied by bystander (B), survival related to shockability of initial rhythm (C), and survival related to presence of return of spontaneous circulation prior to hospital arrival (D) in hospitalized patients after out-of-hospital cardiac arrest CPR - cardiopulmonary resuscitation; ROSC - return of spontaneous circulation

Out of all OHCA patients, 54 (20.8%) survived to discharge from hospital treatment, of which all survived 30-days, except in one case with missing data (Figure 4A). Significantly better survival of patients after OHCA until discharge depending on the initial cause was observed in patients with respiratory etiology of OHCA and only in comparison with other non-cardiogenic causes (all non-cardiogenic causes except respiratory causes and trauma) [p = 0.028; relative risk (RR) 0.388] (Figure 4B). In relation to whether the collapse was witnessed or not, the survival was also significantly higher if there was a bystander (p = 0.033) and with a more pronounced impact if the witness was an EMS mobile team (p = 0.013), although the survival in relation to whether the witness was a bystander or an EMS mobile team was not significantly different (Figure 4C). CPR attempt had an impact on significantly better survival rate in these patients (p = 0.032) (Figure 4D) which was also the case in relation to whether bystander initiated CPR in a group of patients in whom bystanders witnessed CA (p=0.046) (Figure 5A). A similar effect was observed in cases when chest compressions only were applied (p = 0.028) (Figure 5B). Additionally, shockable initial rhythm significantly affected the occurrence of death outcome (p = 0.012) (Figure 5C) and the same result was observed with the influence of any form of ROSC prior to hospitalization (p < 0.01) (Figure 5D). Similar findings of predictive values of analyzed factors on death outcome occurrence in OHCA patients were observed by using Cox regression analysis (Table 2).

 

**Table 2:** Cox proportional hazard model for death outcome during hospitalization HR – hazard ratio; CI – confidence interval; CPR – cardiopulmonary resuscitation; OHCA – out-of-hospital cardiac arrest; EMS – emergency medical service; ROSC – return of spontaneous circulation

Predictor	p value	HR	95% CI
Number of inhabitants less than 100,000	0.292	1.168	0.875-1.558
Female gender	0.382	0.878	0.657-1.174
Day time of OHCA	0.751	0.943	0.657-1.354
Out of residence as an OHCA place	0.167	0.810	0.601-1.092
OHCA witnessed by bystander	0.033*	0.664	0.456-0.968
OHCA witnessed by mobile EMS team	0.013*	0.560	0.355-0.883
CPR initiation	0.032*	0.606	0.384-0.957
Bystander CPR initiation	0.046*	0.492	1.007-2.257
Chest compressions only in bystander CPR	0.028*	0.364	1.054-2.538
Dispatcher assistance to bystander CPR	0.625	0.900	0.728-1.696
Shockable rhythm	0.012*	0.657	0.474-0.912
Achieved any ROSC	˂0.01*	0.304	0.217-0.426

The number of hospital days in hospitalized OHCA survivors was influenced only by the occurrence of any form of ROSC prior to hospitalization with high statistical significance (p < 0.01), while the presence of bystander CPR and shockable initial rhythm were observed as predictors of surviving 30-day period after OHCA event (p < 0.01; RR 2.985 and p < 0.01; RR 4.871, respectively), showing that shockable initial rhythm increased chance of 30-day survival nearly five times, while the absence of bystander-CPR led to the three-fold increase of that chance.

## Discussion

The Serbian healthcare system is similar to the healthcare systems present in many (mostly European) countries, however with several specific characteristics. The basis for prehospital emergency patient care in Serbia consists of EMSs organized according to the Social Health Insurance Model [8]. All citizens have compulsory health insurance which is free for children, retired, and unemployed citizens. Emergency medical care is free for all citizens as well as for all individuals in the Serbian territory holding a temporary or permanent residence permit. In Serbia, the standard for the EMS system is one medical doctor, one nurse/technician, and one driver per 30,000-35,000 citizens 24 hours/day, seven days/week [9]. EMS system in Serbia is organized as field service, through the presence of EMSs covering individual municipalities. Each municipality has its own EMS, including cities consisted of multiple municipalities. EMSs are engaged according to the established priority regime defining emergency orders. The functioning of the healthcare system in Serbia (including emergency medical care) is the responsibility of the Ministry of Health.

Regarding OHCA, there are no official protocols in Serbia defining the process of providing CPR measures. However, measures applied by doctors and other medical team members are based on current ERC guidelines [10]. Taking into consideration emergency healthcare organizations in Serbia, some results of our study are similar to those observed in many other European studies [2,3], however, with some specific aspects. 

Almost all studies report residence as the most commonplace of OHCA. EuReCa ONE study which included data from 27 European countries reported an overall average of residence as the location of OHCA of 69.4%, ranging between 46.4% and 79.9% [2]. EuReCa TWO studies which included data from 28 European countries stated that residence as a place of the collapse was reported in 70.2% of cases, ranging by countries between 51.0% and 81.3% [3]. Similar results were found in many other studies, including American Heart Association’s (AHA) statistical report in 2019 [11]. Our study’s rate of residence as the place of the collapse of 81.0% in all OHCA patients group and 74.1% in the group where CPR was initiated is, therefore, in accordance with EuReCa TWO study findings and recently published studies.

Results of witnessing rate in CPR-attempted OHCA group of patients (49.6%) is also comparable to the EuReCa program. EuReCa ONE study reported an overall average witnessing rate of 66.1%, with a median of country values of 67.5%, and country values range of 37.4%-93.5% [2], while the EuReCa TWO study reported that witnessing collapse was observed in 66.6% of OHCA events, ranging between 50.8% and 91.8% [3]. AHA reported rate in the United States very similar to our findings (49.0%) [11].

Results of the multinational EuReCa TWO study published in 2020 stated that the bystander CPR rate in Serbia was 13%, which was convincingly the lowest rate of all participating countries [3]. In our study, the value of 10.6% of bystander-initiated CPR in all patients with OHCA of any cause is even lower compared to the average value in European countries of 58% and the maximal observed value of 82.6% [3]. Many other studies also reported higher bystander CPR rates [2,12-15]. In the 2019 AHA statistical report, 36.5%-44.5% of non-professionals in the United States initiated CPR [11]. Our study did not analyze the structure of bystanders and their education in providing basic life support measures, although the reported values by AHA are still notably higher than the values we report in this research.

Any ROSC rate of 21.0% within the group of OHCA patients with CPR attempt observed in this study is comparable to the ROSC rates reported by most of the previously published investigations [2-4,16] However, various findings can be found within the literature regarding rates of shockability of initially detected rhythm with values ranging from 1% to over 50% [2,3,11]. The rate observed in this study correlates with previous reports of analysis in the territory of Serbia describing bystander involvement [17,18].

The rate of survival to hospital discharge of 18.8% in all-cause OHCA patients observed in this study is also comparable to the majority of previously reported rates in recently published studies [2,3], as well as 30-day survival rate which is comparable with the values observed in other countries and reported in ERC Guidelines 2021 [19,20].

Results of the analysis of the Serbian OHCA registry published in 2018 outlined gender, age, and time of delivered first DC (direct current) shock as a predictor of ROSC. Those results also emphasized that there is a 2.8 fold increase in chances to initiate CPR if witnesses are present, but that chance to achieve any form of ROSC is 1.6 times higher if the witness is not present. In that study, males had 1.9 times higher chance to achieve any form of ROSC [21]. Our results, however, show that the occurrence of OHCA outside the patient's place of residence increases the chance of any form of ROSC almost four times, and that presence of shockable initial rhythm increases this chance twice, which is in accordance with other recent studies related to European region [3]. Also, our results show that the absence of providing CPR measures by bystanders in those patients in whom they witnessed OHCA reduces the chance of any ROSC prior to hospitalization by 54%.

The presence of witnesses at the scene increased survival, reducing the probability of death by 54%, which makes CPR initiation, and even application of chest compressions only by bystanders, significant survival predictor, reducing the probability of death after OHCA by 50%. Shockability of initial rhythm reduced that probability by one-third, and the achievement of any form of ROSC prior to hospitalization by over two-thirds.

Recent studies reported somewhat similar results when it comes to predicting the overall survival of OHCA patients. Meta-analysis and systematic review published by Chinese authors in 2020 showed that survival to discharge was more likely among patients after bystander-witnessed or EMS-witnessed OHCA, who received bystander CPR [16]. This and similar findings emphasize the importance of advanced life support measures and intensive care in the overall survival of OHCA patients which certainly must take a place in survival prediction [22].

One important aspect of improving presented predictors is certainly education of health care workers at all levels, the existence of defined job positions, and special protocols for specific education of all employees in EMSs. As dispatchers play a very important role, more than half of European countries implement programs of dispatcher-assisted CPR [3], and some countries in the world have gone quite far in that field, even developing protocols for EMS dispatchers [23]. The education of health professionals is out of much higher importance than it is thought since there are studies reporting serious findings concerning physicians' knowledge and skills in emergency situations [24]. Another inevitable topic is also the education of the population in general since bystander CPR has been shown as a significant epidemiologic factor that predicts survival rate in OHCA patients.

Our results showed that Utstein parameters (cardiac arrest of cardiac cause witnessed by a bystander, bystander-initiated CPR, and the presence of initial shockable rhythm) have a significant influence on survival in OHCA patients. This implies that converting the OHCA event into the Utstein event is out of extreme importance for improving the survival rate. Certainly, shortening reaction time is one of the greatest tools for achieving this goal. The results of our study revealed higher survival in the EMS-witnessed group of OHCA patients compared to the bystander-witnessed group, which additionally confirms time determinant as an important factor for survival. In Serbia, EMSs in different regions and different municipalities are organized depending on the available resources. In some parts of Serbia, there are no home health care services fully developed, and EMSs take the responsibility of home health care, which limits the capacities of EMSs in those areas. Therefore, installation of AED devices in hardly reachable and non-urban regions without implemented EMS centers could be necessary for reducing reaction time. This is also inseparable from the education of the general population in order to use AED devices to their full potential.

Additionally, post-resuscitation care and rehabilitation must not be forgotten and the 2015 and 2021 recommendations of the ERC also draw attention to that issue [25,26]. Additionally, the latest recommendations re-emphasize ROSC as the main factor influencing overall, and especially neurological recovery of patients after OHCA [26].

All these factors are essential for the outcome of patients with OHCA and it is extremely important to organize special and detailed steps towards their improvement, in order to achieve a higher survival rate for these patients and their successful recovery.

The main shortcomings of this study include its observational design and, as a consequence, limited ability to report specific causalities. Furthermore, the number of OHCA patients that were defibrillated by AED was negligible in our population (n=3), so no results could be obtained since this small value makes it impossible to apply reliable adjustments for this variable. Our study did not analyze many other factors found by previous studies as significant predictors, including pharmacologic treatment, variables related to time passed between various stages of patient management, different ways of transport, one-year survival rate, and factors related to the hospitalization of OHCA patients. These variables were not analyzed since currently there is no data describing them in the Serbian OHCA registry and although that fact does not influence the power of the results observed in our study, due to the adjustments applied during the statistical analysis of independent predictors, future studies should certainly include a broader spectrum of variables in their analysis as potential predictors. Finally, some of the results, especially regarding survival rates after hospital admission have somewhat limited validity since some variables have had a significant number of missing values and since there was a discontinuity in data collection in certain geographical study regions. Also, poorly developed and small environments were not included in this study and these aspects should be also improved in future research.

## Conclusions

Bystander CPR initiation and ROSC rate still remain the main concern in OHCA epidemiology in Serbia. The Survival-to discharge rate of hospitalized OHCA patients is comparable to the majority of other recent major reports. Collapse outside of residence place, bystander CPR initiation, and initially detected shockable rhythm has been observed as important predictors of ROSC in OHCA patients. Independent predictors of better survival rate in hospitalized OHCA patients are the presence of a witness, bystander CPR initiation, initial shockable rhythm, and presence of ROSC prior to hospitalization, while the predictive factors of 30-day survival rate in hospitalized OHCA patients are bystander CPR initiation and shockability of initially detected rhythm.

The results implicate that it is necessary to understand what factors can increase witnessing rate, how to increase the frequency of initial rhythm shockability, as well as what are the factors that will increase the participation of bystanders in providing CPR measures.
